# Infectious Diseases and Tropical Cyclones in Southeast China

**DOI:** 10.3390/ijerph14050494

**Published:** 2017-05-07

**Authors:** Jietao Zheng, Weixiao Han, Baofa Jiang, Wei Ma, Ying Zhang

**Affiliations:** 1 Department of Epidemiology, School of Public Health, Shandong University, 44 West Wenhua Road, Jinan 250012, China; zhengjietao001@163.com (J.Z.); hanweixiao123@126.com (W.H.); bjiang@sdu.edu.cn (B.J.); 2 Climate Change and Health Center, Shandong University, 44 West Wenhua Road, Jinan 250012, China; 3 School of Public Health, University of Sydney, Sydney 2006, Australia

**Keywords:** tropical cyclone, infectious diseases, impact, China

## Abstract

Southeast China is frequently hit by tropical cyclones (TCs) with significant economic and health burdens each year. However, there is a lack of understanding of what infectious diseases could be affected by tropical cyclones. This study aimed to examine the impacts of tropical cyclones on notifiable infectious diseases in southeast China. Disease data between 2005 and 2011 from four coastal provinces in southeast China, including Guangdong, Hainan, Zhejiang, and Fujian province, were collected. Numbers of cases of 14 infectious diseases were compared between risk periods and reference periods for each tropical cyclone. Risk ratios (RR*s*) were calculated to estimate the risks. TCs were more likely to increase the risk of bacillary dysentery, paratyphoid fever, dengue fever and acute hemorrhagic conjunctivitis (*ps* < 0.05) than to decrease the risk, more likely to decrease the risk of measles, mumps, varicella and vivax malaria (*ps* < 0.05) than to increase the risk. In conclusion, TCs have mixed effects on the risk of infectious diseases. TCs are more likely to increase the risk of intestinal and contact transmitted infectious diseases than to decrease the risk, and more likely to decrease the risk of respiratory infectious diseases than to increase the risk. Findings of this study would assist in developing public health strategies and interventions for the reduction of the adverse health impacts from tropical cyclones.

## 1. Introduction

Tropical cyclones (TCs) are cyclonic currents that occur in tropical or subtropical ocean, often accompanied by high winds, heavy rain and storm surge [1]. They may also be called hurricanes in the northeastern Pacific, typhoons in the northwestern Pacific, cyclones in the southern hemisphere. China is one of the countries that are most seriously affected by TCs, putting almost all coastal provinces in high risks. The coastal provinces in southeast mainland China that suffer the most from TCs are Guangdong, Hainan, Zhejiang, and Fujian [2].

TCs can increase the risk of some infectious diseases, such as leptospirosis, dengue, malaria, cholera, other infectious diarrhea, acute respiratory infection and pulmonary tuberculosis [3,4,5,6,7,8]. For example, an acute diarrhea outbreak occurred after “Thane” in India [3]. An increasing number of acute gastroenteritis was reported in Kananga, Leyte, where evacuated residents had returned home two days after typhoon Haiyan [9]. The incidence of diarrhea, dysentery and acute respiratory infections also increased after cyclone Nargis [8]. An increase in leptospirosis cases linked to post-cyclone clean-up was observed two weeks after cyclone Bejisa in Reunion Island [10].

The increase in infectious disease transmission and outbreaks after TCs could be associated with the prolonged after-effects, which include displaced people, environment changes, increasing vector breeding sites, high exposure, poor water and sanitation conditions, poor personal hygiene and limited access to healthcare services [11]. TCs can lead to heavy precipitation and floods. The water distribution network and sanitation facilities in affected area can be heavily damaged, leading to contaminated drinking water [12]. Poor sanitation conditions play an important role in the transmission of infectious diseases. In addition, in affected areas where the devastation of infrastructures was profound, medical services could be damaged beyond functionality, leading to limited capacity to identify and/or treat patients [13], resulting in the spread of infectious diseases.

A better understanding of what infectious diseases could be affected by TC may assist local health sector in responding to potential outbreaks and allocating health resources in public health emergencies. However, previous studies were restricted to either one single disease category or conducted in small study areas. There are few studies systematically examining TC-sensitive infectious diseases with no studies reported in China. Current knowledge of TC-sensitive infectious diseases is far from clear. Using data from four coastal provinces with the highest frequencies of landing TCs in southeast China, our study aimed to generate a clear spectrum of TC-sensitive infectious diseases in mainland China.

## 2. Materials and Methods

### 2.1. Study Areas and Study Period

**Figure 1:**
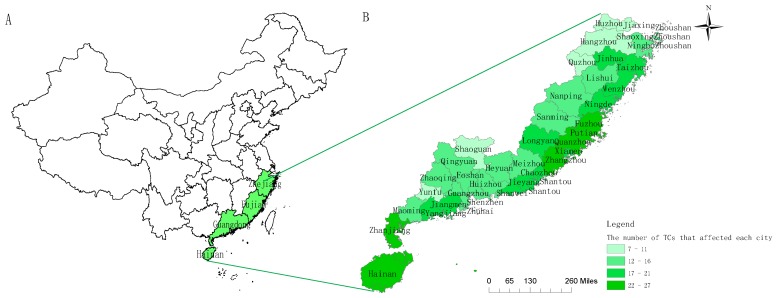
Study areas affected by TCs (tropical cyclones) (**A**) and the number of TCs (cyclone events) that affected each city (**B**), China, 2005–2011.

Four coastal provinces in southeast China, including 42 cities, were the study areas because they were the provinces most hit by TCs (Figure 1A). Our study areas covered a total population of 199.1 million (population of Guangdong, Fujian, Zhejiang, Hainan province was 105.1 million [14], 37.2 million [15], 47.8 million [16], 9.1 million [17], respectively), with 6.8% in Guangdong [14], 8.2% in Fujian [15], 9.6% in Zhejiang [18], and 8.1% in Hainan [17] were older people (≥65 years old). Gross Domestic Product (GDP) in Guangzhou, Fujian, Zhejiang, Hainan province was 775 billion [14], 256 billion [15], 471 billion [16] and 37 billion dollars [17], respectively, in 2011. The climate for each of the four provinces is listed in Table 1. The study period covered the tropical cyclone season (April to October) in the four provinces, from 2005 to 2011.

### 2.2. Data Sources

#### 2.2.1. Disease Data

Case report forms (CRFs) of patients with all national notifiable infectious diseases in the study areas were obtained from the National Notifiable Disease Surveillance System (NDSS) of the Chinese Centers for Disease Control and Prevention (CDC). All eligible cases were reported by local hospitals that received patients. Data from CRFs that were retrieved for this analysis included area code, disease code, case classification, age, gender and date of onset. Travelers who were not local residents were excluded from the analysis.

Currently, there are 39 notifiable infectious diseases in China [25,26]. We excluded the following diseases from our analysis because they are unlikely to be affected by TCs, including sexually transmitted diseases (STD) (i.e., AIDS, gonorrhea, syphilis), blood transmitted diseases (i.e., hepatitis C), rabies, diseases with the shortest incubation period > 45 days (i.e., hepatitis B, echinococcosis, leprosy, filariasis), chronic infectious diseases (i.e., kala-azar, tuberculosis). We also ruled out the diseases whose numbers of reported cases during both disaster and reference periods were less than 10, including plague, cholera, severe acute respiratory syndromes, hepatitis E, poliomyelitis, human avian influenza, epidemic hemorrhagic fever, epidemic encephalitis B, anthrax, amebic dysentery, meningococcal meningitis, pertussis, diphtheria, neonatal tetanus, scarlet fever, brucellosis, leptospirosis, schistosomiasis, epidemic and endemic typhus. We did not include human H7N9 avian influenza as it was not included in the National Notifiable Disease Surveillance System until 2013. Thus, we analyzed 14 infectious diseases including 13 notifiable diseases (bacillary dysentery, typhoid and paratyphoid fever, measles, vivax malaria, nontypeable malaria, dengue fever, influenza and influenza A (H1N1), mumps, rubella, acute hemorrhagic conjunctivitis, infectious diarrhea other than cholera, bacillary and amebic dysentery, typhoid and paratyphoid fever (other infectious diarrhea), hand-foot-and-mouth disease (HFMD), hepatitis A) and varicella because of its high morbidities in the study areas.

#### 2.2.2. Meteorological Data

Daily meteorological data over the study period in the four provinces were obtained from the China Meteorological Data Sharing Service System (http://cdc.cma.gov.cn/). For the cities where no weather station (Huzhou, Dongguan, Zhongshan, Zhuhai, Putian before 2009, Foshan and Chaozhou) was available, data from the nearest station (the distance from the central of the cities to their nearest stations is 18.9–103.2 kilometres) were used. Hainan is a province with a small area of 35.4 square kilometres and it is often affected by TCs as a whole. Thus, we regarded Hainan as one city and the average of the meteorological data from 7 different stations was used to represent its meteorological condition.

#### 2.2.3. Data of TCs

All TCs over the study period that affected the four provinces were included. Basic information of TCs was collected from the Yearbook of Tropical Cyclone provided by the China Meteorological Administration, including grade, landfall, path, time period, the distribution and intensity of precipitation and wind [27].

### 2.3. Statistical Analysis

A single cyclone event can affect several cities and the risk period varied in different cities because of their geographical variations. Therefore, we regarded one single TC that affected N cities as N different cyclone events (CE). For example, if one TC affected 10 cities, it was counted as 10 CEs. We defined that a city was “affected” by TCs if a level 7 wind circle (wind velocity reached 13.9 m/s [28]) of the tropical cyclone hit the city.

We defined the disaster period and reference period with the following considerations. The hazards posed by TCs resulted from three factors, i.e., wind, storm surges and rain. According to previous research on the classification standard of tropical cyclone’s effects [29,30,31,32] as well as our research purpose, we defined a exposure period to TCs as days experienced a tropical cyclone with a level 7 wind circle and above, and satisfied one of the following meteorological conditions (using data from http://cdc.cma.gov.cn): (1) 24 h total rainfall ≥25 mm; (2) a maximum wind speed reached level 6 (wind velocity reached 10.8 m/s); (3) the gust speed reached level 7 (wind velocity reached 13.9 m/s). The disaster periods in this study included the exposure periods to TCs plus the maximum incubation period of each disease. A reference period was defined as days with the same duration and the same days of week as those days of the exposure period, plus one or more weeks before the exposure period. We selected reference period by weeks in order to reach a number of diseases more than zero [5]. The risk ratios (RR*s*) between number of reported cases in the two periods were calculated using the formula: RR = a (number of cases during disaster period)/b (number of cases during reference period) and the 95% confidence interval (CI) of RR = exp (log RR + 1.96$\sqrt{\frac{1}{a}\pm \frac{1}{b}}$) [33]. Assuming that the study population changed little over the course of one season, and by selecting a comparable reference period, the person-times in the denominators of the RRs for the two periods could be equivalent [34,35].

The proportions of TCs that increased the risk of analyzed diseases were compared with the proportions of TCs that decreased the risk using *χ^2^* tests [36]. χ^2^ = (the number of TCs increasing risk −the number of TCs decreasing risk)^2^/(the number of TCs increasing risk + the number of TCs decreasing risk), υ = 1. A disease is considered to be sensitive to TCs if the difference between the two proportions is statistically significant.

### 2.4. Ethical Approval

Disease surveillance data used in this study were obtained from the National Notifiable Disease Surveillance System (NDSS) with the approval by the Chinese Center for Disease Control and Prevention. All identity information of patients was removed before data analysis. The study was approved by the Ethical Review Committee (ERC) of School of Public Health in Shandong University (20120501).

## 3. Results

### 3.1. Basic Information of TCs during the Study Period

From 2005 to 2011, 65 TCs (tropical cyclones) affected Guangdong, Hainan, Zhejiang, and Fujian province (Figure 1A), including 51 in Guangdong, 34 in Hainan, 27 in Zhejiang and 36 in Fujian. The number of TCs that affected each province each year over the study period is shown in Table 2. The number of TCs that affected each city is shown in Figure 1B. We regarded 65 TCs that affected 42 cities as 675 CEs (cyclone events). Detailed information of the 65 TCs is summarized in Supplementary Materials Table S1.

### 3.2. Disease Frequencies in the Study Period

Daily disease frequencies for all analyzed infectious diseases in the study period are summarized separately for cyclone and non-cyclone period in Table 3. We also summarized disease frequencies for low level and high level TCs as well as costal and non-coastal cities respectively, which can be found in Supplementary Materials Tables S2–S5.

### 3.3. Disease Sensitivity to TCs

TCs had different effects on analyzed infectious diseases. TCs were more likely to increase the risk of bacillary dysentery (*p* = 0.001), typhoid fever, paratyphoid fever (*p* = 0.046), other infectious diarrhea, hepatitis A, influenza, influenza A (H1N1), dengue fever (*p* = 0.046) and acute hemorrhagic conjunctivitis (*p* = 0.021) than to decrease the risk, more likely to decrease the risk of HFMD, measles (*p* = 0.005), mumps (*p* < 0.001), rubella, varicella (*p* = 0.013), vivax malaria (*p* = 0.005) and nontypeable malaria than to increase the risk. Effects of TCs on all analyzed infectious diseases are listed in Table 4.

## 4. Discussion

In this study, we examined the sensitivity of a range of infectious diseases to TCs, using data from four provinces that are most affected by TCs in China. We found that TCs are more likely to increase the risk of water-food borne diseases than to decrease the risk. The reason may be that TCs always accompany with strong precipitation which leads to floods and results in drinking water pollution. The reason that TCs are more likely to increase the risk of acute hemorrhagic conjunctivitis than to decrease the risk may be the same. The association between TCs and the increase of bacillary dysentery and other infectious diarrhea was similar to several previous studies in other countries and China. A WHO report shows that diarrhea disease is the major cause of illness after natural disasters (40%) [37]. A study in the U.S. indicates that gastrointestinal diseases were the most commonly recorded acute diseases among evacuees from Memphis and Tennessee after hurricane Katrina in 2005 [38]. In South Korea, during the typhoon periods in 2003, cases of infectious diarrhea hospitalization increased by 46.9% [5]. After typhoon AILA, the incidence of diarrhea significantly increased in two Indian subdivisions in 2009 [39]. A study in Guangdong of China indicated that other infectious diarrhea significantly increased after TCs [40]. A study in Zhejiang found that both typhoons and tropical storms could contribute to an increase in risk of bacillary dysentery and other infectious diarrhea [7].

We also found TCs are more likely to decrease the risk of air borne diseases than to increase the risk. Possible explanations of this may be that respiratory infections mainly spread by droplets. TCs always accompany with high-speed wind, rain and decrease in temperature, which bring air purification to some extent. In addition, TCs will largely reduce the contacts between people, resulting in decreased risk of infectious diseases transmitted through respiratory route. However, TCs can increase the risk of influenza and influenza A (H1N1) (not significantly). The reason might be that the effects of increasing risk factors such as decrease in temperature are larger than the effects of decreasing risk factors such as the purification of air. Among other studies investigating effects of TC on respiratory diseases, a study in Zhejiang of China showed that mumps decreased by up to 79.1% among boys and 75.4% among girls after typhoons [41]. Typhoon Haiyan increased the risk of respiratory illness among children in Canada [42]. Another study in Myanmar showed that the incidence of acute respiratory infections increased after cyclone Nargis [8]. The reason of inconsistent results for respiratory infectious diseases with other studies might be that: (1) most other studies were effects of single TC and the grades of TCs varied; (2) study area and population were different, for example, the increase in the incidence of acute respiratory infections after cyclone Nargis was observed only in children under five years old; (3) seasonal patterns of respiratory infectious diseases were different from our study area.

For mosquito transmitted diseases, TCs are more likely to decrease the risk of vivax malaria than to increase the risk but more likely to increase the risk of dengue fever than to decrease the risk. TCs can decrease the temperature which is unfavorable to mosquito habitat, and the accompanied wind will also influence the flying distance of mosquitoes, but the precipitation will increase the number of mosquito habitat and improve the condition of the habitat, which explains the different risk changes of the two mosquito transmitted diseases. A study in Haiti in the early 1960s indicated that outbreaks of malaria with more than 75,000 cases happened following hurricane Flora and there was an increase of malaria cases in Guatemala and Nicaragua following hurricane Mitch in 1998 [43].

Our study has some advantages. First, using a large population sample, we analyzed impacts of all tropical cyclones affecting the four provinces. Second, we analyzed impacts of TCs on 14 infectious diseases including 13 notifiable infectious diseases, which will provide scientific evidence for taking prevention and control measures after tropical cyclones.

Our study also has some limitations. First, the impacts of TCs on infectious diseases with long lags could be influenced by seasonal effects that could influence transmission of diseases. But we believe the influence of seasonal effects is minimal because seasonal effects would remain constant across the study period. Second, the meteorological factors were not very accurate in seven out of forty-one cities (five cities before 2009 and other two cities) without meteorological stations, but we believe the error is small because we used the data from the nearest stations to represent them. Third, the number of reported cases in reference period was 0 sometimes. So we selected reference period as one or more weeks before the current reference period until the sum of reported cases of the selected reference period was at least one and then average it, which may lead to unstable results (e.g., too wide 95% CIs of RRs).

We believe that our study will contribute to a better understanding of the impacts of TCs on infectious diseases. Health care systems should increase the awareness of the risk of infectious diseases which TCs increase the risk, especially water-food and contact transmitted diseases, and train health professionals to cope with the outbreaks after TCs. Vulnerable people should be taught how to prevent infectious diseases. Drinking water and food hygiene supervision work should be strengthened to prevent infectious diseases after TCs.

## 5. Conclusions

TCs have mixed effects on the risk of infectious diseases, TCs are more likely to increase the risk of intestinal and contact transmitted infectious diseases than to decrease the risk, and more likely to decrease the risk of respiratory infectious diseases than to increase the risk. Findings of this study would assist in developing public health strategies and interventions for the reduction of the adverse health impacts from tropical cyclones.

## Figures and Tables

**Table 1:** The climate of the four provinces.

Climate	Guangdong [19]	Fujian [20]	Zhejiang [21]	Hainan [22]
General condition	It usually has high temperature and rainy days. It has a typical summer season for seven months each year and is one of the rainiest provinces in China.	It has four distinct seasons, with moderate temperature and abundant rainfall.	The same as Fujian.	It has a tropical climate.
Average annual temperature	18–24 °C	17–21 °C	15–18 °C	22.5–25.6 °C
Average annual rainfall	1350–2600 mm	1100–2000 mm	980–2000 mm	1500–2500 mm
The Köppen Climate Classification [23]	Cfa	Cfa	Cfa	Cwa

C: Moist Subtropical Mid-Latitude Climates. Summers: warm and humid, convective thunderstorms; winters: mild, mid-latitude cyclone; Cfa: Summers: hot muggy and frequent thunderstorms. Precipitation is fairly evenly distributed throughout the year. Hurricanes also provide a mechanism for producing precipitation in more tropical regions [24]; Cwa: Summers: hot and humid with intense summer convectional storms. Winter: cold and dry [24].

**Table 2:** The number of TCs that affected each province over the study period.

Province	Year							Total
	2005	2006	2007	2008	2009	2010	2011	
Guangdong	8	8	6	7	10	6	6	51
Fujian	6	5	5	8	4	5	3	36
Zhejiang	5	6	5	4	3	2	2	27
Hainan	4	3	2	3	7	3	5	27

**Table 3:** Average number of cases per day for different infectious diseases in the study period.

Disease	Number of Cases of Disease in Cyclone Period/per Day	Number of Cases of Disease in Non-Cyclone Period/per Day
**Water-Food Transmitted**		
Bacillary dysentery	24.3	21.0
Typhoid fever	4.1	4.5
Paratyphoid fever	8.8	4.1
Other infectious diarrhea	42.4	40.5
Hepatitis A	4.4	3.4
**Air Transmitted**		
Influenza	9.5	7.1
Influenza A (H1N1)	9.5	7.4
HFMD ^a^	67.5	71.0
Measles	8.0	10.8
Rubella	4.7	12.8
Mumps	13.9	18.8
Varicella ^b^	8.3	9.2
**Mosquito Transmitted**		
Vivax malaria	18.2	23.0
Nontypeable malaria	8.9	11.5
Dengue fever	5.6	5.0
**Contact Transmitted**		
Acute hemorrhagic conjunctivitis	69.7	67.1

^a^ Hand, foot, mouth disease, also transmitted through water-food; ^b^ also transmitted through contact.

**Table 4:** Effects of TCs ^a^ on infectious diseases (Total TCs = 675).

Disease	Number of TCs Increasing the Risk (%)	Number of TCs Increasing the Risk with Statistical Significance (%)	Number of TCs Decreasing the Risk (%)	Number of TCs Decreasing the Risk with Statistical Significance (%)	Direction ^d^ of the Effect and *P* ^e^	Range of RR
**Water-Food Transmitted**						
Bacillary dysentery	69 (10.22)	4 (0.59)	36 (5.33)	1 (0.15)	↑0.001	0.47–5.63
Typhoid fever	1 (0.15)	0 (0)	0 (0)	0 (0)	↑0.317	2.38
Paratyphoid fever	4 (0.59)	0 (0)	0 (0)	0 (0)	↑0.046	1.04–6.81
Other infectious diarrhea	246 (36.44)	26 (3.85)	206 (30.52)	19 (2.81)	↑0.060	0.23–14.33
Hepatitis A	4 (0.59)	0 (0)	3 (0.44)	0 (0)	↑0.705	0.75–2.26
**Air Transmitted**						
Influenza	40 (5.93)	8 (1.19)	30 (4.44)	11 (1.63)	↑0.232	0.01–144.38
Influenza A (H1N1)	10 (1.48)	5 (0.74)	8 (1.19)	6 (0.89)	↑0.637	0–10.75
HFMD ^b^	134 (19.85)	26 (3.85)	144 (21.33)	28 (4.15)	↓0.549	0.10–6.73
Measles	18 (2.67)	1 (0.15)	39 (5.78)	4 (0.59)	↓0.005	0.19–3.74
Mumps	84 (12.44)	4 (0.59)	136 (20.15)	21 (3.11)	↓<0.001	0.25–8.17
Rubella	0 (0)	0 (0)	3 (0.44)	2 (0.3)	↓0.083	0.13–0.43
Varicella ^c^	28 (4.15)	2 (0.3)	50 (7.41)	6 (0.89)	↓0.013	0.12–14.24
**Mosquito Transmitted**						
Vivax malaria	0 (0)	0 (0)	8 (1.19)	0 (0)	↓0.005	0.47–0.98
Nontypeable malaria	2 (0.3)	0 (0)	7 (1.04)	1 (0.15)	↓0.096	0.33–1.38
Dengue fever	4 (0.59)	1 (0.15)	0 (0)	0 (0)	↑0.046	1.11–34.38
**Contact Transmitted**						
Acute hemorrhagic conjunctivitis	32 (4.74)	22 (3.26)	16 (2.37)	13 (1.93)	↑0.021	0.18–60.00

^a^ tropical cyclones; ^b^ Hand, foot, mouth disease, also transmitted through water-food; ^c^ also transmitted through contact; ^d^ ↑: more likely to increase the risk than to decrease the risk; ↓: more likely to decrease the risk than to increase the risk; ^e^: The proportions of TCs that increased the risk of analyzed diseases were compared with the proportions of TCs that decreased the risk using *χ^2^* tests.

## Supplementary Materials

The following are available online at http://www.mdpi.com/1660-4601/14/5/494/s1, Table S1: The information of 65 tropical cyclones affecting Guangdong, Hainan, Zhejiang, and Fujian province, Table S2: Effects of low level TCs (tropical depressions and tropical storms) on all analyzed infectious diseases in coastal cities, Table S3: Effects of high level TCs (severe tropical storms and typhoons) on all analyzed infectious diseases in coastal cities, Table S4: Effects of low level TCs (tropical depressions and tropical storms) on all analyzed infectious diseases in non-coastal cities, Table S5: Effects of high level TCs (severe tropical storms and typhoons) on all analyzed infectious diseases in non-coastal cities.
