# Supplementary material for: Infectious Diseases and Tropical Cyclones in Southeast China
Source: Int J Environ Res Public Health. 2017 May 7;14(5):494. doi: 10.3390/ijerph14050494 (PMC5451945; doi:10.3390/ijerph14050494)
Supplement: Supplementary file 1 [file ijerph-14-00494-s001.pdf]

Supplementary Materials: Infectious Diseases and Tropical Cyclones in Southeast China

Jietao Zheng, Weixiao Han, Baofa Jiang Wei Ma and Ying Zhang

Table S1. The information of 65 tropical cyclones affecting Guangdong, Hainan, Zhejiang, and Fujian province.

Year	Dates	The Order Number	Name of TC	Grade of TC	Maximum Wind Speed (m/s)
2005	7.11–7.21	5	Haitang	Typhoon	60
	7.29–8.1	8	Washi	Severe tropical storm	25
	7.30–8.9	9	Matsa	Typhoon	45
	8.10–8.15	10	Sanvn	Severe tropical storm	30
	8.26–9.3	13	Talim	Typhoon	55
	9.5–9.13	15	Khanun	Typhoon	50
	9.15–9.19	16	Vicente	Tropical storm	23
	9.19–9.27	18	Damrey	Typhoon	50
	9.25–10.3	19	Longwang	Typhoon	55
	10.28–11.2	21	Kai-tak	Typhoon	40
2006	5.8–5.19	2	Chanchu	Severe typhoon	50
	6.24–6.29	3	Jelawat	Tropical storm	20
	6.29–7.13	4	Ewiniar	Super typhoon	55
	7.8–7.17	6	Bilis	Severe tropical storm	30
	7.18–7.29	7	Kaemi	Typhoon	40
	7.28–8.5	8	Prapiroon	Typhoon	33
	8.5–8.12	10	Saomai	Super typhoon	60
	8.5–8.11	11	Bopha	Severe tropical storm	25
	9.25–10.2	20	Xangsane	Severe typhoon	50
2007	7.7–7.17	4	Man-yi	Super typhoon	55
	8.5–8.11	7	Pabuk	Severe tropical storm	30
	8.6–8.9	8	Wutip	Tropical storm	20
	8.12–8.24	9	Sepat	Super typhoon	65
	9.15–9.20	13	Wipha	Super typhoon	55
	9.21–9.26	14	Francisco	Tropical storm	20
	9.29–10.5	15	Lekima	Typhoon	33
	10.1–10.10	16	Krosa	Super typhoon	55
2008	4.14–4.20	2	Neoguri	Typhoon	40
	6.18–6.27	7	Fengshen	Severe typhoon	45
	7.13–7.23	8	Kalmaegi	Typhoon	33
	7.24–8.1	9	Fung-wong	Severe typhoon	45
	8.3–8.8	10	Kammuri	Severe tropical storm	25
	8.17–8.23	13	Nuri	Typhoon	40
	9.8–9.22	15	Sinlaku	Super typhoon	55
	9.17–9.26	16	Hagupit	Severe typhoon	50
	9.24–10.5	17	Jangmi	Super typhoon	65
	9.29–10.6	19	Higos	Tropical storm	18
2009	6.17–6.25	3	Linfa	Severe tropical storm	30
	6.22–6.27	4	Nangka	Tropical storm	23
	7.10–7.13	5	Soudelor	Tropical storm	18
	7.15–7.19	7	Molave	Typhoon	38
	7.31–8.9	8	Goni	Severe tropical storm	28
	8.3–8.13	9	Morakot	Typhoon	40
	9.9–9.12	14	Mujigae	Tropical storm	20
	9.13–9.16	16	Koppu	Typhoon	40
	9.25–10.1	17	Ketsana	Typhoon	40
	9.28–10.15	19	Parma	Super typhoon	55

	10.18–10.20	23		Tropical depression	15
**2010**	7.11–7.18	2	Conson	Typhoon	35
	7.17–7.23	3	Chanthu	Typhoon	35
	8.22–8.25	6	Mindulle	Severe tropical storm	30
	8.27–9.4	7	Lionrock	Severe tropical storm	28
	8.29–9.1	9	Namtheun	Tropical storm	18
	9.7–9.14	11	Meranti	Typhoon	35
	9.15–9.22	12	Fanapi	Super typhoon	52
	10.4–10.10	14	TD1002	Tropical depression	15
	10.13–10.24	15	Megi	Super typhoon	72
**2011**	6.9–6.11	6	Sarika	Tropical storm	20
	6.17–6.25	7	Haima	Tropical storm	20
	6.21–6.27	8	Meari	Severe tropical storm	30
	7.25–7.31	11	Nock-ten	Severe tropical storm	28
	7.27–8.9	12	Muifa	Super typhoon	65
	8.21–8.31	15	Nanmadol	Super typhoon	60
	9.23–10.1	21	Nesat	Severe typhoon	45
	9.26–10.5	23	Nalgae	Severe typhoon	50

Table S2. Effects of low level TCs [a] (tropical depressions and tropical storms) on all analyzed infectious diseases in coastal cities.

**Diseases** [b]	**Disease Frequencies in the Disaster Period/per Day × Hundred Million**	**Disease Frequencies in the Reference Period/per Day × Hundred Million**	**RR**	**95% CI**
Water-food transmitted				
Bacillary dysentery	12.64	11.88	1.06	0.56–2.02
Typhoid fever	2.30	4.62	0.50	0.14–1.80
Paratyphoid fever	7.59	4.18	1.82	0.69–4.79
Other infectious diarrhea	29.67	29.38	1.01	0.67–1.53
Hepatitis A	2.30	2.24	1.02	0.23–4.56
Air transmitted				
Influenza	6.41	8.81	0.73	0.32–1.66
Influenza A (H1N1)	9.30	7.45	1.25	0.57–2.73
HFMD [c]	31.01	32.48	0.95	0.64–1.42
Measles	7.91	10.28	0.77	0.36–1.63
Rubella	3.12	8.46	0.37	0.13–1.06
Mumps	8.21	10.21	0.80	0.38–1.70
Varicella [d]	5.73	7.92	0.72	0.30–1.73
Mosquito transmitted				
Vivax malaria	6.07	8.91	0.68	0.29–1.58
Nontypeable malaria	6.99	9.52	0.73	0.33–1.62
Contact transmitted				
Acute hemorrhagic conjunctivitis	65.17	65.31	1.00	0.76–1.32

[a] tropical cyclones; [b] The numbers of reported cases of dengue fever from both disaster and reference periods were less than 10 and were not analyzed; [c] Hand, foot, mouth disease, also transmitted through water-food; [d] also transmitted through contact.

Table S3. Effects of high level TCs [a] (severe tropical storms and typhoons) on all analyzed infectious diseases in coastal cities.

**Diseases** [b]	**Disease Frequencies in the Disaster Period/per Day × Hundred Million**	**Disease frequencies in the Reference Period/per Day × Hundred Million**	**RR**	**95% CI**
Water-food transmitted				
Bacillary dysentery	18.45	16.82	1.10	0.64–1.88
Typhoid fever	3.14	1.32	2.38	0.46–12.41
Paratyphoid fever	5.33	3.30	1.62	0.53–4.93
Other infectious diarrhea	27.56	25.62	1.08	0.70–1.67
Hepatitis A	3.53	2.20	1.60	0.41–6.30
Air transmitted				
Influenza	6.19	5.69	1.09	0.43–2.74
Influenza A (H1N1)	2.39	6.60	0.36	0.11–1.21
HFMD [c]	83.97	88.36	0.95	0.75–1.21
Measles	3.35	4.62	0.73	0.23–2.28
Mumps	10.42	14.17	0.74	0.38–1.41
Varicella [d]	5.70	5.28	1.08	0.41–2.83
Mosquito transmitted				
Vivax malaria	17.96	21.44	0.84	0.50–1.39
Nontypeable malaria	4.75	5.61	0.85	0.31–2.29
Contact transmitted				
Acute hemorrhagic conjunctivitis	11.76	18.91	0.62	0.34–1.12

[a] tropical cyclones; [b] The numbers of reported cases of dengue fever and rubella from both disaster and reference periods were less than 10 and were not analyzed; [c] Hand, foot, mouth disease, also transmitted through water-food; [d] also transmitted through contact;.

Table S4. Effects of low level TCs [a] (tropical depressions and tropical storms) on all analyzed infectious diseases in non-coastal cities.

**Diseases** [b]	**Disease Frequencies in the Disaster Period/per Day × Hundred Million**	**Disease Frequencies in the Reference Period/per Day × Hundred Million**	**RR**	**95% CI**
Water-food transmitted				
Bacillary dysentery	34.13	28.39	1.20	0.58–2.48
Paratyphoid fever	14.32	2.10	6.81	0.84–55.57
Other infectious diarrhea	48.95	48.47	1.01	0.57–1.80
Air transmitted				
Influenza	20.08	12.89	1.56	0.57–4.30
Influenza A (H1N1)	22.60	2.10	10.75	1.39–83.43
HFMD [c]	59.10	62.81	0.94	0.56–1.58
Measles	14.61	20.42	0.72	0.27–1.89
Mumps	23.57	31.73	0.74	0.34–1.61
Varicella [d]	16.04	15.98	1.00	0.37–2.74
Mosquito transmitted				
Dengue fever	11.69	10.51	1.11	0.33–3.72
Contact transmitted				
Acute hemorrhagic conjunctivitis	194.33	155.03	1.25	0.92–1.70

[a] tropical cyclones; [b] The numbers of reported cases of typhoid fever, hepatitis A, rubella, vivax malaria and nontypeable malaria from both disaster and reference periods were less than 10 and were not analyzed; [c] Hand, foot, mouth disease, also transmitted through water-food; [d] also transmitted through contact.

Table S5. Effects of high level TCs [a] (severe tropical storms and typhoons) on all analyzed infectious diseases in non-coastal cities.

Diseases [b]	Disease Frequencies in the Disaster Period/per Day × Hundred Million	Disease Frequencies in the Reference Period/per Day × Hundred Million	RR	95% CI
Water-food transmitted				
Bacillary dysentery	71.41	56.77	1.26	0.76–2.08
Other infectious diarrhea	125.27	117.05	1.07	0.74–1.54
Air transmitted				
Influenza	19.72	0.69	28.13	0.89–890.07
Mumps	33.87	48.36	0.7	0.37–1.32

[a] tropical cyclones; [b] The numbers of reported cases of typhoid fever, paratyphoid fever, hepatitis A, influenza A (H1N1), HFMD, measles, rubella, varicella, vivax malaria, nontypeable malaria, acute hemorrhagic conjunctivitis and dengue fever from both disaster and reference periods were less than 10 and were not analyzed.

© 2017 by the authors; licensee MDPI, Basel, Switzerland. This article is an open access article distributed under the terms and conditions of the Creative Commons by Attribution (CC-BY) license (http://creativecommons.org/licenses/by/4.0/).
